# Tonotopically Arranged Traveling Waves in the Miniature Hearing Organ of Bushcrickets

**DOI:** 10.1371/journal.pone.0031008

**Published:** 2012-02-13

**Authors:** Arun Palghat Udayashankar, Manfred Kössl, Manuela Nowotny

**Affiliations:** AK Neurobiologie and Biosensorik, Institute of Cell Biology and Neuroscience, J.W. Goethe University, Frankfurt, Germany; Claremont Colleges, United States of America

## Abstract

Place based frequency discrimination (tonotopy) is a fundamental property of the coiled mammalian cochlea. Sound vibrations mechanically conducted to the hearing organ manifest themselves into slow moving waves that travel along the length of the organ, also referred to as traveling waves. These traveling waves form the basis of the tonotopic frequency representation in the inner ear of mammals. However, so far, due to the secure housing of the inner ear, these waves only could be measured partially over small accessible regions of the inner ear in a living animal. Here, we demonstrate the existence of tonotopically ordered traveling waves covering most of the length of a miniature hearing organ in the leg of bushcrickets *in vivo* using laser Doppler vibrometery. The organ is only 1 mm long and its geometry allowed us to investigate almost the entire length with a wide range of stimuli (6 to 60 kHz). The tonotopic location of the traveling wave peak was exponentially related to stimulus frequency. The traveling wave propagated along the hearing organ from the distal (high frequency) to the proximal (low frequency) part of the leg, which is opposite to the propagation direction of incoming sound waves. In addition, we observed a non-linear compression of the velocity response to varying sound pressure levels. The waves are based on the delicate micromechanics of cellular structures different to those of mammals. Hence place based frequency discrimination by traveling waves is a physical phenomenon that presumably evolved in mammals and bushcrickets independently.

## Introduction

Georg von Békésy was awarded the Nobel Prize in 1961 for his discovery of traveling waves in the cochleae of human cadavers [1]. Subsequently the existence of traveling waves in vivo has been shown indirectly in a wide range of species. Recent studies [2], [3] have shown that insects use traveling waves for frequency discrimination. Traveling waves are also the primary mechanism behind passive tuning in the cochlea (see detailed review by Robles *et al.* [4]. Thomas Gold [5] hypothesized the existence of an active process in the ear as early as 1948, but it took more than three decades to provide experimental evidence [6] for his findings. A broad consensus has emerged now, that active and passive processes come together to achieve the sensitivity, frequency tuning and selectivity displayed by the ear, but questions related to the way these are implemented by the micromachinery in the ear remain unanswered [7]. The coiled shape and tough access to the cochlea impede measurements, and a common solution is to break open the *scala tympani* in the basal turn and measure the basilar membrane response there [8]–[11]. This leads to measurements that are quite restricted in space so that only limited *in-vivo* data are available from other turns of the cochlea that process important behaviourally relevant frequencies in mammals.

**Figure 1:**
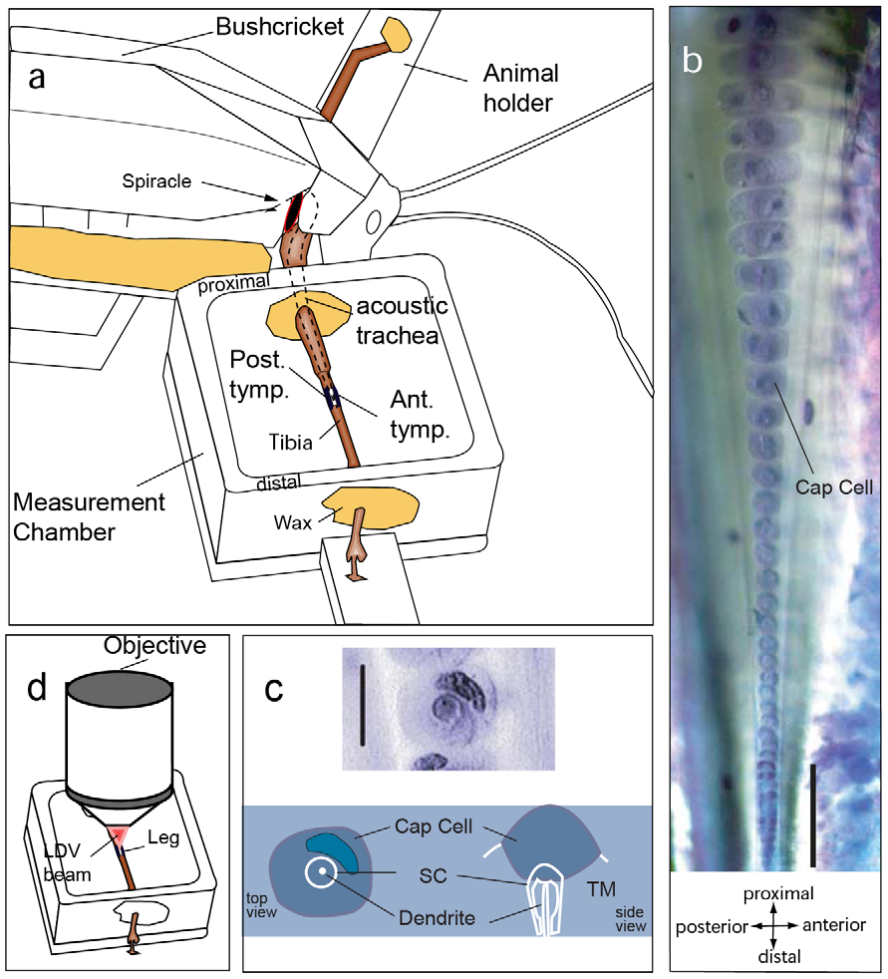
Measurement setup and preparation of the *crista acustica*. (**a**) The bushcricket is waxed onto a freely movable stage and the leg is passed though a chamber filled with insect Ringer and is fixed with wax to avoid movement during the measurement. (**b**) *Crista acustica* stained with methylene blue as viewed with a 10× objective. Notice the gradual decrease in dimensions of the cap cells from proximal to distal region of the leg. (**c**) Magnified view of a cap cell and scolopidium, as viewed with a 40× objective. The bottom panel shows schematic drawings of the cap cell as viewed from the top and side, not to scale. Abbreviations: TM - tectorial membrane, S.C. - scolopidium (**d**) A laser Doppler vibrometer coupled to a confocal microscope was used to make the vibration measurements. The laser beam was focussed on the scolopidia through a small window opening above the organ location. Scale bars: (b): 100 µm and (c): 10 µm.

Tropical bushcrickets (*Mecopoda elongata*) offer several advantages over mammals in that their high frequency (6 to 80 kHz) hearing organ is located in the foreleg, protected only by a thin layer of cuticle, and thus is easily accessible. Furthermore, the short length (∼1 mm) and the lack of pronounced coiling makes up to 70% of the organ visible under the microscope. Sound excites the organ by two pathways. The primary input is through the spiracle, a small opening located in the thorax of the animal where sound enters the acoustic trachea (Fig. 1a). The horn shaped acoustic trachea of *M. elongata* [12] acts as a high pass filter and provides an amplification of approximately 20 dB [13]. Finally, sound energy is transmitted to the part of the acoustic trachea that is lined dorsally with the hearing organ, known as the *crista acustica* (CA). This part of the trachea (dorsal wall) is tapered in width like the mammalian basilar membrane. The second acoustic input pathway is through the tympana, which are compliant plate-like structures abutting the acoustic trachea on the anterior and the posterior sides. Sound impinges directly on the tympana and alters pressure in the acoustic trachea, leading to excitation of the organ, particularly at low frequencies (4–24 kHz) [14].

The CA is made of about 47 distinct receptor cell complexes, called auditory sensillae. in *M. elongata*. Theses cell complexes consist of a bipolar sensory neuron and a set of supporting cells, including a scolopale and a cap cell. The cap cells (stained in Fig. 1b, c) are graded in size from the proximal to the distal region. Both the width of the cap cells and that of the underlying trachea decrease along the distal extension of the CA. Each cap cell is associated with a scolopale cell and the dendrite of a distinct sensory neuron (Fig. 1c). The transduction channel is believed to be located near the tip of the sensory dendrite [15]. Mechanical excitation of the sensory cell complex leads to the transduction [16]. The auditory sensillae are held in place by an overlying membrane, known as the tectorial membrane (TM). The TM possesses bands running parallel to the length of the CA that support the cap cells on the anterior and posterior sides [17].

Sound leads to the stimulation of sensory cells and the position of the stimulated cells depends on the applied frequency. Such a place based frequency representation is known as tonotopy. Tonotopy of the bushcricket *crista acustica* in *Mygalopsis marki* was first mapped by Oldfield using intracellular recordings of the sensory neurons [18]–[20]. However, the mechanical basis of which, remains unclear and speculative [21], [22]. In order to elucidate the mechanical basis of the tonotopy we used laser Doppler vibrometry to measure the frequency dependent response of the *crista acustica* to acoustic free field stimulation.

## Results

### The hearing organ in bushcrickets (*crista acustica*) can be readily accessed in their forelegs

Sound enters the bushcricket ear through a spiracle and travels along a horn shaped acoustic trachea (Fig. 1a). A middle ear like in mammals is absent. In bushcrickets, the dorsal wall of the acoustic trachea shows a systematic variation in width along the organ with the low frequency region being wider (Fig. 1b) and the gradient in scolopidial cap cell dimension provides a mass gradient along the organ. However, unlike the basilar membrane in mammals that is surrounded by fluid on both sides, the CA is loaded with fluid on top and air below (see Fig. S1).

**Figure 2:**
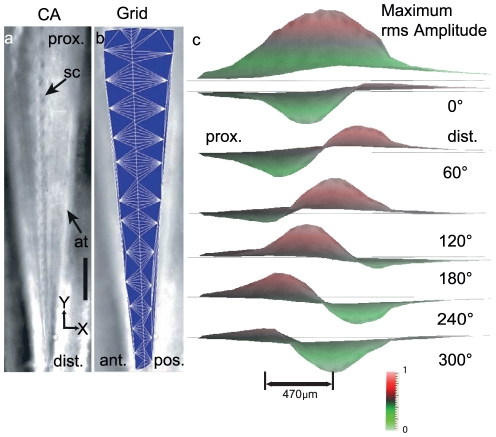
Traveling waves along the *crista acustica*. (a) Typical preparation of the *crista acustica* after removing the overlying cuticle, tissue and hemolymph as seen with a 10× objective. (b) Measurement grid used for the measurements. Nodes on the graph represent the points scanned by the laser-Doppler-vibrometer. The edges of the grid were determined by an automated nearest neighbour algorithm built into the measurement software to interpolate the measured points by triangulation to render the surface data. (c) Side view of the interpolated surface measured for stimulus frequency of 12 kHz at 80 dB SPL. The top most panel indicates the maximum RMS amplitude measured at each point during the entire cycle. The subsequent panels indicate the flow of traveling wave from the distal (dist.) to the proximal (prox.) location with time for one period in 60° steps. Increase of the sound frequency leads to a gradual distal shift of the area responding with the maximum rms velocity. The colour coded scale bar indicates the normalized velocity range. Abbreviations: ant. - anterior; at.- acoustic trachea; ca- *crista acustica*; dist.- distal; post. – posterior, prox.- proximal; sc- scolopidium; Scale bar: 100 µm.

To get access to the CA we opened a small window above of the high frequency hearing organ in bushcrickets i.e. the CA as visualized in Fig. 1b. The preparation (seen in Fig. 2a) was placed in ringer solution [23] and was bathed in the solution between measurements. However, during the experiment the ringer solution was removed using a piece of tissue paper while leaving a mound above the organ. This ensured the undamped movement of the two tympana during the experiment while bathing the organ with fluid. Distortion Product Otoacoustic Emission (DPOAE) thresholds measured before and after opening the window were rather stable and only slightly increased by 4 dB below 10 kHz and 6 dB above 10 kHz (Figure S2). We do not expect these small DPOAE changes to have a significant influence on the data reported in this study.

We used a scanning vibrometer coupled to a confocal microscope to measure the sound-induced motion at different points on the CA (see nodes in Fig. 2b). Edges were drawn up between the nodes by the measurement software in order to interpolate and render surface data. Traveling waves became apparent as slow moving waves that travel along the length of the CA in the proximal direction (Fig. 2c). The above figure shows the progress of the traveling wave along the hearing organ for one sine wave cycle measured at 12 kHz.

### Traveling waves propagate in the direction opposite to stimulation in the *crista acustica*

In bushcrickets, in contrast to mammals, sound enters the hearing organ at the proximal part of the leg, where low frequencies are represented. However, the sound-induced traveling waves travel, as in mammals from the high frequency region (distal part) towards the low frequency region (proximal part). Thus in bushcrickets, similar to mammals traveling waves are not comparable to surface waves. Rather, they are generated by a smooth gradient in mechanical properties whose propagation velocity and wavelength at any given location depends on the local mechanical properties.

**Figure 3:**
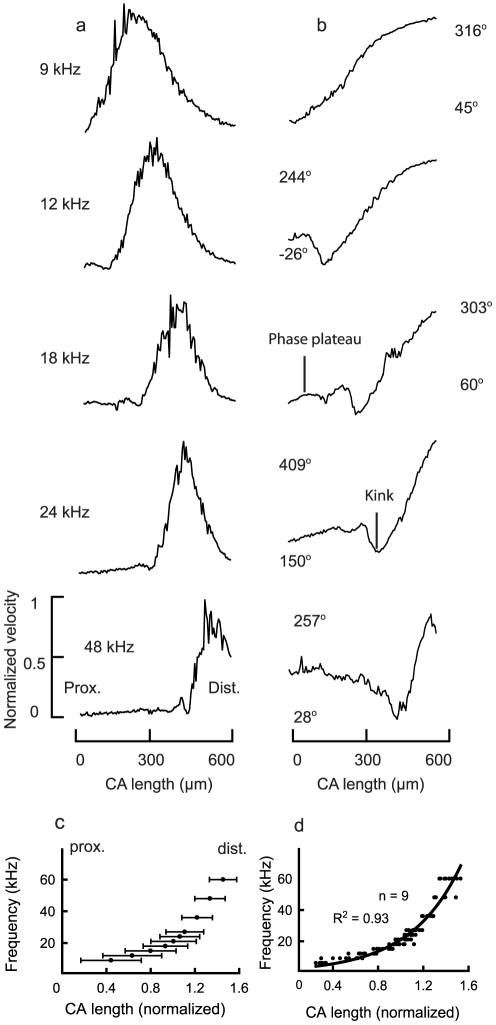
Tonotopic representation of pure tone stimulated motion of the *crista acustica*. (a) Normalized mechanical response profiles of the *crista acustica* along the dendrite axis measured for various frequencies (9–48 kHz) at 80 dB SPL. (b) Corresponding phase responses to a. The values above and below each curve are the maximum and minimum values of the measured phase response. (c) Spatial spread (the width of the response peak at the magnitude 5 dB below the maximum velocity) for a given frequency calculated from the velocity profiles for various frequencies (9–60 kHz) in one preparation. The spatial spread (horizontal lines) and the corresponding midpoints (dots) are marked in the figure. (d) Distribution of midpoints of spatial spread (maximum velocity) along the length of the organ induced by different frequencies. Data points from nine different animals were fitted with an exponential function.

Notice a gradual build up in the amplitude of the traveling wave as it progresses along the CA. Figure 2c shows in 60° steps the progress of the wave through the CA for the entire sine wave cycle. Here a stimulus at 12 kHz and 80 dB SPL was used. Only 70% (∼700 µm) of the organ can be seen at once due to the objective used (see Methods). The wavelength was computed to be ∼470 µm at the CF location. This explains why just over a single wavelength is necessary for the traveling wave to reach the CF location. The build up until the CF location is gradual (w.r.t. distance) but the attenuation thereafter is rather fast and the proximal slope is much sharper than the distal slope (Fig. 3a). An animation of the profile and motion of the traveling wave from the distal (high frequency) to the proximal (low frequency) part of the organ are shown in Videos S1 and S2.

### Tonotopy in the *crista acustica* is exponential

In bushcrickets, for the first time we were able to map a tonotopic representation by direct mechanical measurements along the length of the hearing organ. The frequency dependent velocity profiles are shown in Fig. 3a. With increasing stimulus frequency, the maximum velocity response shifts towards the distal part of the leg. Furthermore, the spread of the region responding with maximum velocity decreases from low to high frequencies. This is expected given the decrease in dimensions of the *crista acustica* from the proximal to the distal part of the leg. Upon analysis of the phase response, it becomes clear that the total phase lag, which is the difference between maximum and minimum of the phase profile, exceeded 210° at all tested stimulus frequencies (Fig. 3b), e.g. at 21 kHz the total phase accumulation was 256°±6.2° (n = 6). Since the maximum phase lag characteristic of a first order resonator would be only 90°, the large phase accumulation along the CA indicates considerable longitudinal coupling. Consistent with the traveling wave originating at the distal end of the CA, an increase in phase lag can be noticed up until the CF location. At the proximal end of the bulge in the amplitude response, a pronounced kink appears. This can also be seen in the phase response (Fig. 3b). Further, the *crista acustica* motion is asymmetrically tuned around the characteristic frequency location with a steeper slope towards the proximal end (see amplitude profiles around CF location in Fig. 2c and Fig. 3a).

In order to quantify the tonotopy along the CA, we plotted the normalized spatial spread of the maximum |V_rms_| response corresponding to each frequency against the length of the CA (Fig. 3c). The extent of the spatial spread decreases from the proximal to distal part of the CA. This correlates well with the change in dimensions of the cap cells and the acoustic trachea along the length of the organ. The mid points of the spatial extension of frequency-specific CA motion (black dots in Fig. 3c) from different measurements are fitted by an exponential function (Fig. 3d) according to the equation:
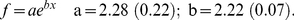
(1)Here the coefficients a and b were determined by nonlinear regression with the values in brackets representing the standard error. The R^2^ value for this fit was 0.93.

### Propagation velocity and wavelength of traveling waves in bushcrickets are similar to those of mammals

**Figure 4:**
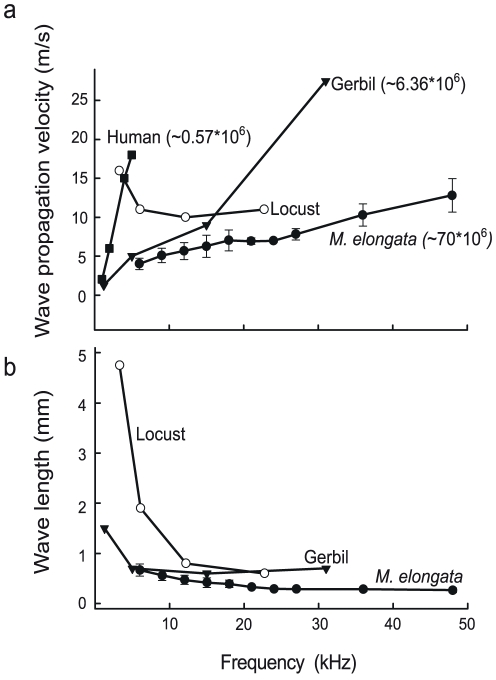
Comparative traveling wave characteristics. (a) Mean traveling wave velocity for increasing sound frequencies in *M. elongata* compared to other species. Notice that the slope for each species (except in locusts) is inversely proportional to it's corresponding topographic spread (in Hz/m, indicated in brackets; see text for explanation). Humans have the smallest topographic spread (indicative of an excellent frequency resolution) (b) The corresponding wavelength values to a. Notice that for the bushcrickets and mammals the wavelength values are in the same range for the shown frequency range. Since velocity data for the human cochlea were determined by indirect means, it was not possible to get the corresponding wavelength data.

Velocity and wavelength of propagation are the most important parameters for traveling wave characterization and they can be calculated from the phase response (2). We compared the frequency dependence of the propagation velocity and wavelength derived from the phase responses across humans [24], gerbils [25] locusts [2] and bushcrickets (Fig. 4a and b). As no direct measurement for humans are available, the velocity and wavelength data were deducted indirectly from auditory brain stem responses. In the bushcricket, traveling wave velocity increases from 4 to 12 ms^−1^ for an increase in frequency from 6 to 48 kHz. However, the slope is much smaller than that of mammals. In contrast to the velocity increase with rising frequency found in mammals and bushcrickets, in locusts there is an overall decrease of velocity. The wavelength decreased for the same frequency range from 0.67 mm to 0.27 mm. If we define topographic spread (ts) as:
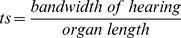
(2)the corresponding values for humans, gerbils and *M. elongata* may be approximated to be 0.57×10^6^, 6.36×10^6^ and 70×10^6^ Hz/m respectively. Notice that the slope of traveling wave velocity vs. frequency curve is inversely proportional to the topographic spread along the cochlea (Fig. 4a) [2].

### Growth in the amplitude response is non-linear

**Figure 5:**
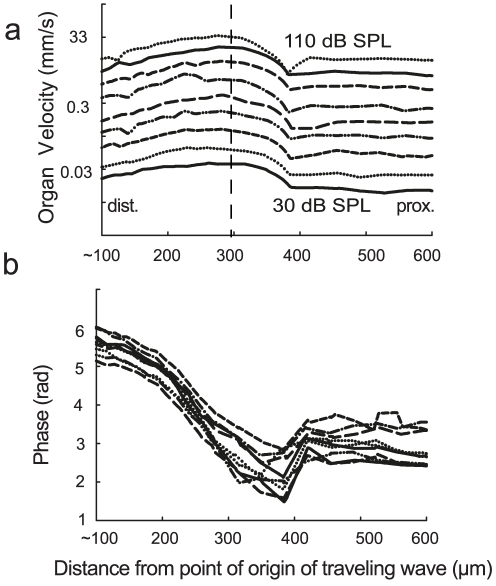
Compressive nonlinearity of the velocity response in *M. elongata*. (a) The velocity response profiles for various sound pressure levels (30–110 dB SPL) at 21 kHz. An increase in sound pressure level leads to a nonlinear of the velocity response. This effect is particularly apparent above 80 dB SPL. (b) The phase response profiles for the same sound pressure levels as in a. The dotted line represents the centre frequency location.

When assessing the dependence of CA motion on sound pressure level within a level range of 30–110 dB SPL (Fig. 5a), a slight compression of the velocity response becomes apparent. The compression is largest at high sound pressure levels and at the characteristic frequency location (in Fig. 5a at ∼300 µm). It reaches a value of ∼73 dB for an 80 dB increase in sound pressure level i.e. the compression was 7 dB. In addition, in the bushcricket the mechanical response is less sensitive than mammals. The threshold sound pressure level necessary to reach a velocity of 1 µms^−1^ was 30 dB SPL. The phase profiles show clustering at the distal end, while spreading out at the proximal end (Fig. 5b).

## Discussion

It was shown in the present paper that bushcrickets exhibit frequency dependent traveling waves. Direct mapping of tonotopy using traveling waves has not been possible so far in mammals due to anatomical limitations mentioned earlier. It has only been done through indirect methods [26]–[28] in mammals. Our preparation of the bushcricket did not present us with these limitations.

Despite the differences between the vertebrate hearing organ (located in the skull) and the *crista acustica* (found in forelegs) of bushcrickets both share similarities. Presumably in both types of organs, the smooth gradients in mechanical properties lead to traveling waves along the organ that are tonotopically ordered. In comparative biology, this kind of similarity in function is termed as analogous, since it evolved independently in the two organisms without a near common ancestor. It may be interesting however, to note in this light, that other vertebrates like lizards seem to lack traveling waves [29]. Another important similarity between bushcrickets and mammals is the presence of a tectorial membrane that covers the hearing organ. A recent study [30] showed how the tectorial membrane provides longitudinal coupling in the cochlea. The bushcricket tectorial membrane could provide longitudinal coupling within the hearing organ, as demonstrated for the mammalian tectorial membrane [30].

Despite the functional similarities, there are pronounced differences between the two organs. One of them concerns how the sound waves reach the hearing organ. Even though bushcrickets possess a trachea that acts as a high pass filter and boosts a certain frequency range like the ear canal in mammals, they lack a middle ear. But that is not surprising as sound is not transmitted from one medium to another like in the cochlea. The most striking difference however, is in the anatomical specialization of the bushcricket hearing organ namely the tympana. In the case of *M. elongata*, there are two tympana on the leg surface that are associated with each CA. These are complaint plate-like structures. Further, in *M. elongata* the tympana boost lower frequencies relevant for the communication among these insects [28].

Von Békésy performed experiments in model cochleae where, unlike the *in-situ* case, he delivered the stimulus to the apex (low frequency region). However, the traveling wave direction remained the same i.e. it propagated from the base to apex [31]. This points to the fact that the direction of the traveling wave is a more fundamental physical effect emerging from a smooth gradient in the mechanical properties in a specific direction and may explain why traveling waves emerging from the high frequency region are the dominant mechanism underlying frequency discrimination in a series of longitudinally coupled resonators. Further evidence comes from the observation that in almost all mammals and insects where traveling waves were reported so far, they commence at the high frequency region independent of the stimulus location [2]–[4].

The idea of a reverse traveling wave has been put forth in the cochlea when explaining the mechanism underlying distortion product generation [32]. Such an interference of forward and reverse traveling waves leads to a distinct kink in the phase and amplitude response [33]. A similar effect can also be noticed in the bushcricket data (Fig. 3a and b). Notice that one can see a kink in the amplitude and phase response that is proximal to the point of maximum response. One possible explanation for the kink comes from transmission line theory. It is known that when the transmission line is not impedance matched, reflections of the traveling waves are possible. This could be the case in bushcrickets where the distal end of the acoustic trachea seems to merge into a thin tube running down the tibia and possibly not being impedance matched. However, an alternative interpretation of the kink could be that the kink is a result of the cancellation of slow traveling waves and fast compression waves e.g. [26], [34], [35].

The local resonance frequency (*f*r) of any auditory partition is determined by the its mass (m) and stiffness (s), *f*r = ½π √s/m. The idea of local resonances has been invoked previously to explain traveling waves in the cochlea [36]. It is indeed plausible that the local mass and stiffness parameters in bushcrickets vary adequately in order to give rise to the tonotopy seen in these hearing organs. One study [37] where the stiffness gradient of the cochlear partition was measured in the apical turn inferred that the gradient in fact may be enough to support the local resonance idea in the cochlea. However, another study, where wave number measurements in the basal turn were done [38] cast doubts on the above idea despite finding a considerable mass gradient.

We compared the traveling waves and the nonlinear compression in bushcrickets with mammalian data. For the gerbil, Ren [11] measured over a distance of ∼600 µm, using a stimulus frequency of 16 kHz, a wave length of ∼200 µm and a wave velocity of 3.2 m/s. This compares quite well with our data. We measured the wavelength and velocity to be 317 µm and 6.7 m/s respectively for a 21 kHz stimulus over an observed organ length of ∼700 µm in the bushcricket. Since the length of our preparation is comparable to that of the traveling wave wavelength we could only visualize one wave length of the traveling wave for a given sine wave cycle. However, in Ren's study [11] it was possible to visualize a little more than one wavelength for a sine wave cycle.

By changing the sound pressure level of the stimulus, nonlinear compression of the velocity response can be measured. In bushcrickets it was found to be about 7 dB for an 80 dB increase in SPL. Comparable measurements in gerbils [11] have reported a much higher compression of 35 dB for an 80 dB stimulus range. Further, to evoke a velocity response of 1 µm/s in the gerbil basilar membrane, a stimulus of only 10 dB SPL was required in comparison to 30 dB SPL in the case of the bushcricket CA. This could be due to the level dependant active electromechanical amplification in the cochlea e.g. [39]. In comparison to mammals, no broadening of the velocity profile with increase in sound pressure level towards the proximal (low frequency) region was found in bushcrickets [40]. However, a clustering in phase responses towards the high frequency region could be seen.

It was reported that other insects also use traveling waves for frequency discrimination. Windmill et. al [2] measured traveling waves on the tympanum of locusts, short-horned grasshoppers that have their sensory cells located directly beneath the tympanum, and concluded that these waves were phenomenologicaly similar to those of mammals despite the absence of any hydrodynamic interaction with the membrane. Laser vibrometry measurements on the cicada tympanum [3] revealed that these insects also exhibit traveling waves. Traveling wave velocity decreases with increase in frequency for locusts [2], remains constant for the cicada [3] and increases in the case of mammals and bushcrickets. Traveling wave wavelength decreases with increase in frequency for all the above species considered.

If the traveling wave indeed leads to the opening of mechano-electrical transduction channels, the found sound-induced mechanical response of the hearing organ should match the electrophysiological data. Stölting *et al.* [40] reported a linear relationship between the measured characteristic frequency and the cap cell number using single cell recordings. We obtained an exponential relationship while plotting the maximum velocity response for different frequencies against length of the *crista acustica*. However, taking into account the fact that cap cells determine the spacing of sensory cells along the organ and increase in size towards the proximal location (see Fig. 1b) our results are in line with the electrophysiological findings.

An exponential tonotopy has also been determined by indirect means in other hearing organs [26]–[28]. In the case of some mammals [41] it has been demonstrated that in the apical portion of the cochlea low frequencies are compressed i.e. they occupy shorter basilar membrane lengths compared to higher frequencies in the basal portion (see Eq.1 of Robles et al.[4]). We obtained a similar result in bushcrickets, with a smaller slope in the low frequency area (6–24 kHz) compared to the high frequency (>24 kHz) area (Fig. 3d). An interesting observation regarding the tonotopy characteristic is that if we fit the low frequency part of the tonotopy plot in Fig. 3d with a linear function of shallow slope and the high frequency with a steeper slope, both functions would meet approximately at the centre of the hearing organ (∼18 kHz).

Given that the properties of the traveling waves are similar to those found in mammals, our study complements space-limited data obtained from mammalian preparations and opens up new possibilities to perform *in-vivo* experiments in the bushcrickets. Further, we show the presence of nonlinear compression in the velocity response. Thus bushcrickets could be an interesting model system to put to test theories underlying the physics of cochlear traveling waves and DPOAE generation [42].

## Materials and Methods

### Animal preparation

The insect species used in all experiments described here belong to the tropical bushcricket species *Mecopoda elongata* (Orthoptera; Tettigoniidae Phaneropterinae). These were bred in our in-house facility in Frankfurt am Main. For the preparation, the animals were anesthetized with CO_2_. Their wings, mid-legs and hind-legs were clipped off. They were then fixed onto a freely movable metal platform with rosin-beeswax (Fig. 1a). A sharp razor blade was used to open a small window (length of ∼700 µm) above the organ. The overlying tissue and the hemolymph were carefully removed with a fine pair of tweezers to expose the organ. Insect ringer, prepared according to Fielden [23] was used to fill the chamber (Fig. 1b) immediately following the opening of the organ in order to prevent the preparation from drying out. However, during measurement in order to enable the tympana to move freely, the ringer solution was carefully drained out of the chamber with a piece of tissue paper while maintaining a mound of solution on top of the organ. This introduced a time limit as we needed to take care that the ringer solution did not evaporate during the course of the measurement.

### Mechanical measurements

A Laser Doppler Vibrometer (LDV) scanning system (Polytec MSV-300, Waldbronn, Germany) was used for the mechanical measurements. The scan head (OFV-534) of the system was coupled to a microscope (Axio Examiner, Zeiss, Germany) via a microscope adapter (OFV-072, Polytec, Germany). The resulting laser spot below the used 10× objective (N-Acroplan, Zeiss, Fig. 1d) had a diameter of <10 µm and was positioned under video control (VCT-101). With the objective used, it was possible to scan 70% of the length of the organ at once. The *crista acustica* displacements induced by our range of stimulus frequencies were within this area. Once the focus was set for optimal reflection of light from the cap cells, it was kept unchanged during the entire course of the experiment.

### Sound stimulus calibration and generation

The function generator (NI 611×) of the LDV software was used to generate pure sine wave tones in the following steps: 9, 12, 15, 18, 21, 24, 27, 36, 48 & 60 kHz. The sound pressure levels were calibrated with an acoustic calibrator (Brüel & Kjaer 2610). The generated signal was fed into a stereo power amplifier (Rotel Electronics RB 850) and attenuator (Hewellet Packard 350D) before being fed to the loudspeaker (Technics EASTH 400A). At the used stimulus frequencies, the frequency response of the system was flat (±5 dB). The loudspeaker was placed at a distance of 23 cm to the preparation such that the ribbon of the loudspeaker was in a plane perpendicular to the spiracle.

### Data evaluation

Post processing of the signal from the LDV was done with software provided by Polytec (PSV 8.6, Polytec GmbH, Waldbronn, Germany). The sampling rate of the signals was 256 kHz. Sets of 50 data windows of 16 ms duration were acquired and averaged for each grid point. Both displacement and velocity information were simultaneously acquired during the experiment. A rectangular FFT (Fast Fourier Transform) function was used to produce the frequency spectrum for the data collected from each location. The resolution of this spectrum was 62.5 Hz and a bandwidth of 1–100 kHz was produced. The FFT data was used to calculate magnitude and phase of the velocity responses. By combining data from the point measurements on the given preparation, surface data were produced for the relevant frequencies. Coherence between the measured laser signal and a reference beam was calculated at every point for the relevant frequencies. Only those points that had a coherence of above 90% were considered valid (Fig. S3).

### Quantification of tonotopy

We quantified the tonotopy across several preparations. For each profile a line was drawn at 5 dB below the maximum (normalized to 1 ms^−1^), defining the spatial spread of sound-induced response. The spatial spread was plotted against the location of the *crista acustica* (seen in Fig. 2a). The mid point of each spatial spread is also marked in the figure. Notice that in order to average the data across the preparations we had to normalize the X-axis. This was necessary, as it was difficult to define a single reference point common to all preparations. In order to do this we chose the mean location of the special spread mid points from each animal. For our measurements (9–60 kHz) this mean location i.e. x = 1 lay between 21–24 kHz for all the data sets. The other locations were then normalized to the mean location. Statistical analyses were performed with Matlab 2009b (Mathworks, USA) and SigmaPlot 10 (Systat Software, USA).

## Supporting Information

Figure S1 **Comparative schematic of the cross section of the mammalian cochlea (a) and bushcricket** ***crista acustica*** (b). a) Cross section of the mammalian cochlea. The three fluid filled chambers are indicated, namely the *scala vestibuli*, *scala media* and *scala tympani*. The *scala media* contains the organ of Corti (oC). The basilar membrane is formed at the interface between the *scala tympani* and *scala media*. b) Cross section of the front leg of the bushcricket at the tympanal organ location is shown. The two tympana, namely the anterior (ant.) and posterior (post.) tympana are indicated. The *crista acustica* is surrounded by air below and hemolymph above.
(EPS)

Figure S2 **Difference in DPOAE (2f1-f2) thresholds measured using a −10 dB threshold criterion before and after removing the cuticle overlying the** ***crista acustica*** **.** The figure shows DPOAE thresholds differences plotted against frequency for 2f1-f2. Notice that for frequencies <10 kHz the differences are within ±5 dB. For this frequency range threshold difference was 4.3±3.8 dB. However, at frequencies >10 kHz the differences were higher 6±3.4 dB.
(EPS)

Figure S3 **Coherence Maps of the LDV measurement** a. Here the reference signal against which coherence is calculated is shown. b. This figure shows measured velocity response to a pure tone of 15 kHz, 80 dB SPL. c. Here the coherence value calculated for the given measurement is shown. Notice that the coherence value for the entire area is above 0.9. Maximum value the coherence signal can take is 1. The deviation from 1 represents the sum of relative input noise power and relative output noise power.
(EPS)

Video S1 A time animation of the *crista acustica* (CA) response for one sine wave cycle described in Fig. 3 is shown. The stimulus frequency used was 12 kHz at 80 dB SPL. The motion of the CA from the top, side and a perspective view can be seen. As the traveling wave along the longitudinal axis from the distal end of the leg to the proximal end, it displaces the organ presumably leading to transduction. The surface animation (see Methods) was created after applying a median filter to the raw data.
(MOV)

Video S2 The top panel shows a top view of the *crista acustica* response shown in Fig. 3. The line in the centre lies on the scolopidial axis. The bottom panel shows an animation the velocity profile for one sine wave cycle (12 kHz, 80 dB SPL) taken along the line indicated in the top panel. Notice that as the traveling wave progress along the organ, tiny peaks begin to emerge at the organ location excited by the traveling wave at any given instant. Further, the asymmetry related to the peak of the traveling wave can be seen i.e. the wave builds up slowly until the CF location (∼200 µm) and falls off rapidly beyond the CF location.
(MOV)

## References

[1] Von Békésy G (1960). Experiments in Hearing..

[2] Windmill JF, Göpfert MC, Robert D (2005). Tympanal traveling waves in migratory locusts.. J Exp Biol.

[3] Sueur J, Windmill JF, Robert D (2006). Tuning the drum: the mechanical basis for frequency discrimination in a Mediterranean cicada.. J Exp Biol.

[4] Robles L, Ruggero MA (2001). Mechanics of the mammalian cochlea.. Physiol Rev.

[5] Gold T (1948). Hearing. II. The Physical Basis of the Action of the Cochlea.. Proceedings of the Royal Society, Series B, Biological Sciences.

[6] Kemp DT (1978). Stimulated acoustic emissions from within the human auditory system.. J Acoust Soc Am.

[7] Ashmore J, Avan P, Brownell WE, Dallos P, Dierkes K (2010). The remarkable cochlear amplifier.. Hear Res.

[8] Ruggero MA, Rich NC, Recio A, Narayan SS, Robles L (1997). Basilar membrane responses to tones at the base of the chinchilla cochlea.. J Acoust Soc Am.

[9] Russell IJ, Nilsen KE (1997). The location of the cochlear amplifier: spatial representation of a single tone on the guinea pig basilar membrane.. Proc Natl Acad Sci U S A.

[10] Rhode WS, Recio A (2000). Study of mechanical motions in the basilar membrane of the chinchilla cochlea.. J Acoust Soc Am.

[11] Ren T (2002). Longitudinal pattern of basilar membrane vibration in the sensitive cochlea.. Proc Natl Acad Sci U S A.

[12] Nowotny M, Hummel J, Weber M, Möckel D, Kössl M (2010). Acoustic-induced motion of the bushcricket (*Mecopoda elongata*, Tettigoniidae) tympanum.. J Comp Phys A.

[13] Hoffmann E, Jatho M (1995). The acoustic trachea of Tettigoniids as an exponential horn: Theoretical calculations and bioacustical measurements.. J Acoust Soc Am.

[14] Hummel J, Kössl M, Nowotny M (2011). Role of the tympanum in the hearing of bushcrickets.. J Exp Biol.

[15] French AS (1988). Transduction mechanisms of mechanosensilla.. Ann Rev Entomol.

[16] Oldfield BP, Hill KG (1986). Functional organization of insect auditory sensilla.. J Comp Phys A.

[17] Schumacher R (1975). Scanning-electron-microscope description of the tibial tympanal organ of the Tettigonioidea (Orthoptera, Ensifera).. Z Morphol Tiere.

[18] Oldfield BP (1982). Tonotopic organisation of auditory receptors in Tettigoniidae (Orthoptera: Ensifera).. J Comp Physiol.

[19] Oldfield BP (1984). Physiology of auditory receptors in two species of Tettigoniidae (Orthoptera: Ensifera).. J Comp Physiol.

[20] Oldfield BP (1985). The role of the tympanal membranes in the tuning of auditory receptors in Tettigoniidae (Orthoptera: Ensifera).. J Exp Biol.

[21] Bangert M, Kalmring K, Sickmann T, Stephen R, Jatho M (1998). Stimulus transmission in the auditory receptor organs of the foreleg of bushcrickets (Tettigoniidae) I. The role of the tympana.. Hear Res.

[22] Yack JE (2004). The structure and function of auditory chordotonal organs in insects.. Microsc Res Tech.

[23] Fielden A (1960). Transmission through the last abdominal ganglion of the dragonfly nymph, Anax imperator.. J Exp Biol.

[24] Serbetçioğlu MB, Parker DJ (1999). Measures of cochlear traveling wave delay in humans: I. comparison of three techniques in subjects with normal hearing.. Acta Otolaryngol.

[25] Olson ES (1999). Direct measurement of intra-cochlear pressure waves.. Nature.

[26] Tsuji J, Liberman MC (1997). Intracellular labeling of auditory nerve fibers in guinea pig: central and peripheral projections.. J Comp Neurol.

[27] Müller M (1996). The cochlear place-frequency map of the adult and developing mongolian gerbil.. Hear Res.

[28] Vater M, Kössl M (2011). Comparative aspects of cochlear functional organization in mammals.. Hear Res.

[29] Peake WT, Ling A (1980). Basilar-membrane motion in the alligator lizard: Its relation to tonotopic organization and frequency selectivity.. J Acoust Soc Am.

[30] Ghaffari R, Aranyosi AJ, Richardson GP, Freeman DM (2010). Tectorial membrane traveling waves underlie abnormal hearing in Tectb mutant mice.. Nat Commun.

[31] Von Békésy G (1928). Zur Theorie des Hörens; die Schwingungsform der Basilarmembran.. Phys Z.

[32] de Boer E, Zheng J, Porsov E, Nuttall AL (2008). Inverted direction of wave propagation (IDWP) in the cochlea.. J Acoust Soc Am.

[33] Dong W, Olson ES (2008). Supporting evidence for reverse cochlear traveling waves.. J Acoust Soc Am.

[34] Cooper NP, Rhode WS (1996). Fast travelling waves, slow travelling waves and their interactions in experimental studies of apical cochlear mechanics.. Aud Neurosci.

[35] Rhode WS (2007). Basilar membrane mechanics in the 6–9 kHz region of sensitive chinchilla cochleae.. J Acoust Soc Am.

[36] Bell A (2004). Hearing: travelling wave or resonance?.. PLoS Biol.

[37] Emadi G, Richter CP, Dallos P (2004). Stiffness of the gerbil basilar membrane: radial and longitudinal variations.. J Neurophysiol.

[38] de La Rochefoucauld O, Olson ES (2007). The role of organ of Corti mass in passive cochlear tuning.. Biophys J.

[39] Johnstone BM, Patuzzi R, Yates GK (1986). Basilar membrane measurements and the traveling wave.. Hear Res.

[40] Ren T, He W, Gillespie PG (2011). Measurement of cochlear power gain in the sensitive gerbil ear.. Nat Commun.

[41] Stölting H, Stumpner A (1998). Tonotopic organization of auditory receptors of the bushcricket Pholidoptera griseoaptera.. Cell and Tissue Research.

[42] Kössl M, Möckel D, Weber M, Seyfarth E-A (2008). Otoacoustic emissions from insect ears: evidence of active hearing?.. J Comp Phys A.

